# Numerical values modulate size perception

**DOI:** 10.3758/s13414-024-02875-w

**Published:** 2024-04-19

**Authors:** Aviv Avitan, Dror Marom, Avishai Henik

**Affiliations:** 1 Department of Psychology, Ben-Gurion University of the Negev, P.O.B. 653, Beer-Sheva, Israel; 2 The Zelman Center for Brain Science, Ben-Gurion University of the Negev, Beer-Sheva, Israel

**Keywords:** Numerical processing, Magnitude interaction, Size-congruity effect (SiCE), Mental representations, Point of subjective equality

## Abstract

The link between various codes of magnitude and their interactions has been studied extensively for many years. In the current study, we examined how the physical and numerical magnitudes of digits are mapped into a combined mental representation. In two psychophysical experiments, participants reported the physically larger digit among two digits. In the identical condition, participants compared digits of an identical value (e.g., “2” and “2”); in the different condition, participants compared digits of distinct numerical values (i.e., “2” and “5”). As anticipated, participants overestimated the physical size of a numerically larger digit and underestimated the physical size of a numerically smaller digit. Our results extend the shared-representation account of physical and numerical magnitudes.

## Introduction

Processing of various magnitudes and their interactions has been investigated for decades (e.g., Meck & Church, 1983). A typical demonstration of magnitude interaction is the size-congruity effect (SiCE; Besner & Coltheart, 1979). The SiCE denotes that numerical judgements of digits are performed faster and more accurately when their physical sizes are congruent with their numerical values (e.g., 3 6) than when they are incongruent (e.g., 3 6). Importantly, a SiCE is typically also observed when digits are compared based on a physical aspect. For instance, size judgements of digits are faster and more accurate for congruent than incongruent pairs (Henik & Tzelgov, 1982).

### The locus of magnitude interaction

To deepen our understating of magnitude interaction, their locus has been investigated. Their locus is of interest because it may illuminate how our cognitive system integrates information from different channels of magnitude. In the case of the SiCE, two prominent accounts were put forward: the shared-representation account and the shared-response account. The shared-representation account suggests that information from both channels of magnitude (i.e., physical and numerical magnitudes) converge on a single a-modal representation whose outputs are further processed (Schwarz & Heinze, 1998). In contrast, the shared-response account suggests that information from both channels are processed in parallel by separate mechanisms that interact at the response selection stage (Otten et al., 1996; Santens & Verguts, 2011). Interestingly, studies in support of each account coexist in the literature. Indications of early magnitude interaction during stimulus-evaluation stages support the shared-representation account (Cohen Kadosh & Henik, 2006, 2008; Schwarz & Heinze, 1998). Moreover, this account resonates with theories of a shared brain locus for magnitude processing in the intraparietal sulcus (IPS; Cohen Kadosh et al., 2005; Fias et al., 2001; Walsh, 2003). Other studies support the shared-response account (Cohen Kadosh et al., 2011; Sobel et al., 2016). For instance, Cohen Kadosh et al. (2011) showed that response suppression, rather than suppression of magnitudes, modulated the SiCE for physical comparative judgements (Cohen Kadosh et al., 2011). To recapitulate, the interaction of physical and numerical magnitudes of digits in either loci (stimulus-evaluation stages or response-related stages) may underlie the SiCE. In fact, the locus of their interaction was shown to depend on task requirements (Cohen Kadosh et al., 2007).

### Departure from common experimental designs

The SiCE is commonly studied using chronometric measures with Stroop-like tasks (e.g., the numerical Stroop; Henik & Tzelgov, 1982). To study further the SiCE, some researchers departed from common experimental designs that were used hitherto. Studies that used visual search tasks revealed that numerical information was extracted rapidly both when it was relevant (Corbett et al., 2006) and when it was irrelevant (Krause et al., 2017) to the task. Moreover, it was shown that numerical information of digits guided visual search, in addition to their physical information (i.e., the physical similarity of digits; Godwin et al., 2014; Schwarz & Eiselt, 2012). For instance, Godwin et al. (2014) observed that distractor digits of close numerical values (i.e., whose numerical distance to the target digit was small) were more likely to be attended to than digits of distant numerical values. More recently, it was shown that congruent digits were classified more easily within a size category than incongruent digits due to higher sensitivity (in terms of signal detection theory; Reike & Schwarz, 2017). To illustrate, exemplars of the digit “2” were classified more successfully based on their physical size within a set of small sizes (i.e., numerical value is congruent with size category) than exemplars of the digit “9” (numerical value is incongruent with size category). This observation was taken to support the shared-representation account and indicate that the internal size representation of a digit may be modified by its irrelevant numerical magnitude.

### The current study

Motivated by evidence of early perceptual manifestations of the SiCE – the current study used two psychophysical experiments to quantify how numerical and physical magnitudes of digits interact. Participants were presented with two same (e.g., 2-2) or different (e.g., 2-5) digits that differed in physical sizes. They were asked to select the physically larger digit while ignoring the numerical values of the digits. Next, we computed the point of subjective equality (PSE) of the two digits. PSE is the size at which the two digits were perceived as equal. Note that this novel experimental approach to the SiCE has two main advantages over previous studies. First, this approach studies perceptual sensitivity mechanisms directly, whereas RT-based approaches study the contribution of these mechanisms indirectly. Furthermore, this novel approach goes beyond indicating early perceptual effects to provide quantification of these effects. This second advantage would allow the shared-representation account to delineate how (rather than merely propose that) outputs from different channels of magnitude converge on a unified mental representation. In turn, the current approach would lead to a more nuanced shared-representation account of our symbolic numerical system. Investigations within a more nuanced shared-representation account could challenge (or substantiate) existing, RT-based theoretical ideas regarding our symbolic numerical system. For instance, representation-focused investigations of individuals with developmental dyscalculia (i.e., studies of their representations of numerical magnitude) may challenge existing RT-based reasoning on the matter (e.g., Ashkenazi et al., 2009).

## Experiment 1

### Method

#### Participants

We calculated a priori the statistical power to detect with 80%, a medium-large-size effect (Cohen’s $d$ between 0.7 and 0.8; Cohen, 1988). This effect size was chosen as, generally speaking, effects in psychophysical experiments are large. Our a priori analysis suggested a total sample size ranging from 15 to 19 participants. We recruited 17 participants (11 females, mean age = 27.64 years, *SD* = 2.54) from the BGU participant pool. Participants received monetary reward for their participation. All participants had normal or corrected-to-normal vision. All participants signed a consent form prior to their participation in the experiment. Participants were debriefed in the end of the experiment.

#### Stimuli

Stimuli were pairs of digits written as in a stopwatch (i.e., where 2 is a mirror image of 5). In Experiment 1, the pairs we used were 2-2 and 2-5. The standard digit was always “2”. The size of the standard digit was held constant throughout the experiment and subtended a visual angle of 1.94 ^∘^. The size of the reference digit (i.e., the other digit that is not the standard digit) was manipulated throughout the experiment. We used 10 sizes for the reference digit: there were five smaller sizes (i.e., 1.81^∘^, 1.84^∘^, 1.86^∘^, 1.89^∘^, 1.92^∘^), one size that was identical to the standard digit (i.e., 1.94^∘^) and four larger sizes (i.e., 1.97^∘^, 2.0^∘^, 2.03^∘^, 2.06^∘^) than the size of the standard digit.^1^

#### Design and procedure

The current study was programmed and run via OpenSesame version 3.3.14 for Windows (Mathôt et al., 2012). The monitor resolution was 1920 x 1080. The study was run on a Windows 10 operating system. The participants carried out the experiment in a dimly illuminated room, seated exactly 55 cm from the computer monitor. They were instructed to maintain fixation on the fixation point throughout the experiment. Participants rested their heads on a chin-rest throughout the experiment. Participants were instructed to choose the physically larger digit in the pair as accurately as possible. They were instructed to ignore any other aspect of the digits (e.g., the numerical values of the digits). The experiment had two conditions: an identical condition and a different condition. In the identical condition, participants were presented with the pair 2-2 (an identical numerical value). In the different condition, participants were presented with the pair 5-2 (a different numerical value). Importantly, we used the digits “2” and “5” written as in a stopwatch to match the perceptual attributes of the stimuli as much as possible. Note that “2” and “5” share the same number of line strokes (five strokes) and are configured in a similar fashion (i.e., each is a mirror image of the other). Previous studies that sought to match physical attributes of digits also used the digits “2” and “5” (e.g., Corbett et al., 2006). All participants carried out both conditions in separate blocks. The order of conditions in the experiment was counterbalanced across participants. Each trial began with the presentation of a fixation point at the center of the screen for 500 ms. Next, a pair of digits was presented for another 500 ms. Note that we limited stimuli presentation time to increase the likelihood of participants to process the irrelevant numerical magnitude (Experiment 4 in Corbett et al., 2006, showed that unlimited presentation time debilitated the rapid extraction of numerical magnitude). The centers of the presented digits were 1.84^∘^ left and right of center. Participants could respond from the onset of the digits and up to 4,000 ms after their onset. Participants were instructed to press the “C” key to choose the digit on the left and the “M” key to choose the digit on the right. For each condition, there were four experimental blocks. In each experimental block, all standard-reference size combinations were repeated eight times, except for the sizes 1.84^∘^ and 2.0^∘^ that were repeated 16 times.^2^ These combinations were presented randomly to the participants. Thus, each experimental block consisted of 96 trials. The target digit (i.e., the physically larger digit) was presented left of center in half of the trials and right of center in the other half of trials. In total, all standard-reference size combinations were repeated 32 times in each condition (except for the sizes 1.84^∘^ and 2.0^∘^ that were repeated 64 times). Altogether, there were 384 trials for each condition (four blocks, with 96 trials each) and 768 trials in the entire experiment (two conditions, with 384 trials each).

### Results

**Fig. 1:**
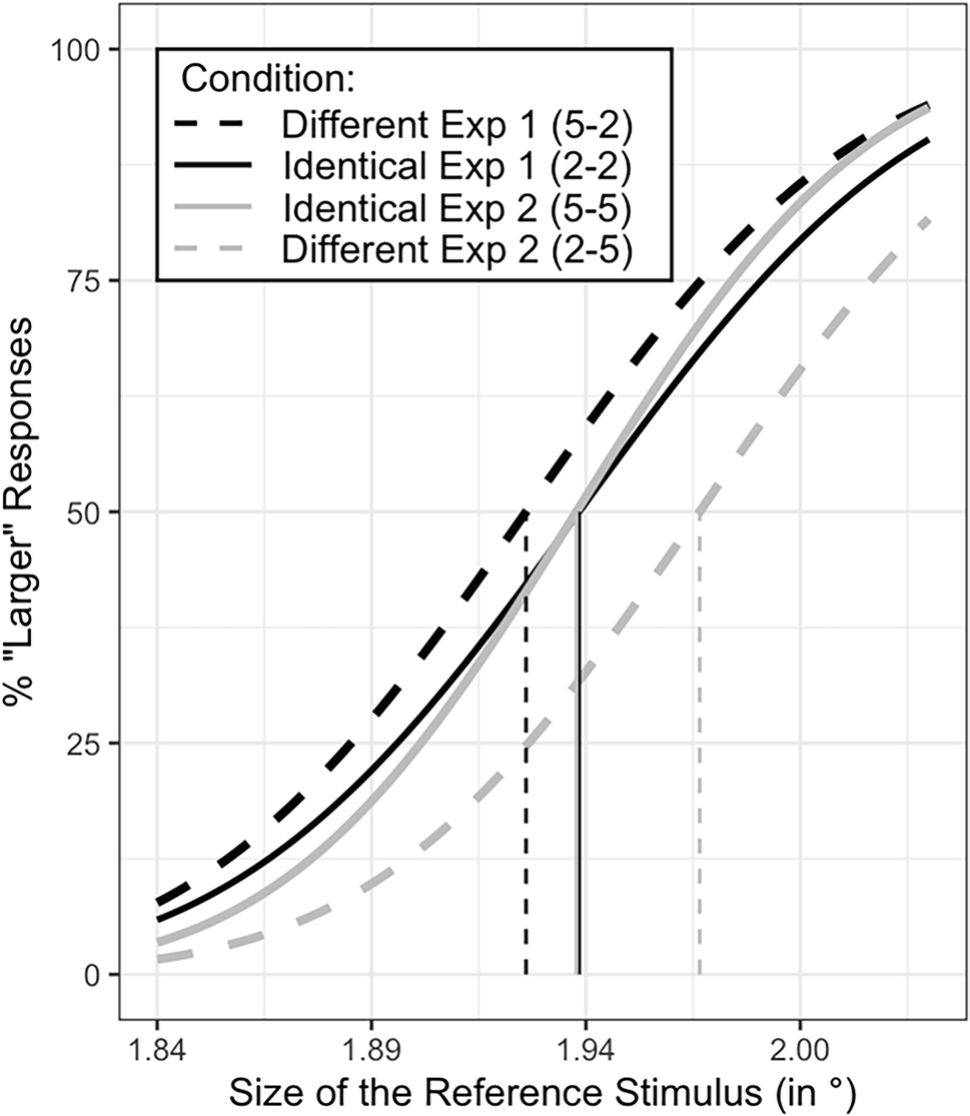
Psychophysical functions of our experimental conditions pooled across participants. The legend denotes the condition. The left digit in legend labels denotes the reference digit in each condition (e.g., “5” was the reference digit in the different condition in Experiment 1). The X-axis denotes the size of the reference digit in visual angles. The Y-axis denotes the probability to respond “larger” to the reference digit. Vertical lines denote the point of subjective equality (PSE) values of each condition. See the Appendix for the raw (unmodeled) subject mean for each condition overlayed on the model curves

**Fig. 2:**
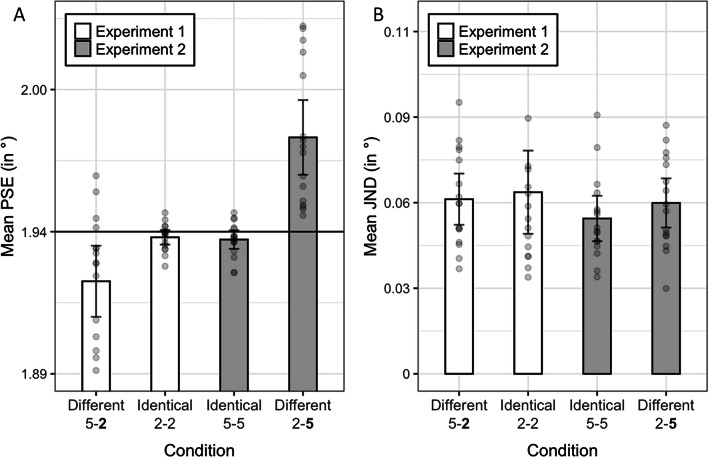
Point of subjective equality (PSE) (**Panel A**) and just noticeable difference (JND) (**Panel B**) means of our experimental conditions pooled across participants. The legend denotes the experiment. The X-axis denotes the condition and the pair of digits that was used. The bold digit in the first and fourth values on the X-axis denotes the standard digit in these conditions (i.e., the digit whose size was held constant). The Y-axis denotes mean PSE values (Panel A) and mean JND values (Panel B) in visual angles. Error bars denote 95% CIs. The gray points denote individual values of participants. The horizontal black solid line in Panel A denotes the size of the standard digit

One participant was excluded from analyses because he failed to comply with task instructions. Thus, analyses were performed on data of 16 participants. First, we excluded trials in which participants failed to respond (consisting of 1.3% of all trials). Next, we calculated for each of the reference stimuli the proportion of trials in which it was perceived as larger than the standard stimuli. This calculation was performed for each participant, in each condition (i.e., for the identical condition and for the different condition). We then fitted the data to a sigmoid function that plotted these proportions against size values ranging from 1.81^∘^ to 2.06^∘^. To validate the fit of these responses to a sigmoid function, we computed Goodness of Fit (GOF) scores for each condition, for each participant, by calculating its r^2^ values. An exclusion cutoff of GOF < 0.7, in either of the conditions, was predetermined. This GOF cutoff was used in similar studies (e.g., Zitron-Emanuel & Ganel, 2018). None of the remaining 16 participants were excluded due to low GOF scores. The total averages (*SD*s) of GOF scores were 0.95 (0.04) and 0.95 (0.02) for the identical and different conditions, respectively. We calculated the PSE and JND of each participant, in each condition (we used *MixedPsy* R package for analyses and plotting; Moscatelli & Balestrucci, 2017). PSEs were calculated by computing the value that corresponds to 50% correct discrimination. At this value, participants perceived the reference digit as equal in size to the standard digit. JNDs were calculated by computing the distance between the values corresponding to 25% and 75% correct discrimination, divided by two. PSE (or JND) values that deviated by 2.5 *SD*s (in either direction) from the group’s mean was predetermined as another exclusion cutoff. None of the participants were excluded due to deviant PSE (or JND) values. We conducted two t-tests to examine the difference between the PSE means and the JND means. We found that the mean PSE in the identical condition was significantly larger than the mean PSE in the different condition, $95\% {\text{CI}}\left[0.08, 1.15\right], t\left(15\right)=2.45, p=.026 , d=.63.$ In contrast, JND means were not significantly different between conditions ($t\left(15\right)=0.63, p=.53$). The difference between PSE means in Experiment 1 is illustrated in Fig. 1 (compare the black solid and the black dashed psychophysical functions) and in Fig. 2, Panel A (compare the white bars).

### Interim discussion

Results of Experiment 1 indicated that the irrelevant numerical magnitude of the digits indeed permeated perception of physical magnitudes. This is indicated by the leftward shift of the PSE in the different condition from the PSE in the identical condition (see Figure 1 for illustration of this shift). To interpret, the digit “5” was perceived as equal in size to the digit “2” when presented slightly smaller (in physical, objective terms) relative to an identical “2”. We did not observe a significant difference between JND values. This is not surprising, as JND values in perceptual estimations are related to the range of stimulus sizes used in the experiment (Namdar et al., 2018). In our case, the range of stimulus sizes was identical across experimental conditions. Therefore, JND measures are not expected to vary between conditions. To interpret in terms of channels of magnitude, the channel of physical magnitude needed a slightly smaller input (i.e., a physically smaller size) to match a “5” with a “2”. This smaller input from the physical channel compensated for the larger input that was fed forward by the channel of numerical magnitude (i.e., the numerical magnitude of “5”). Note, that this interpretation is consistent with the shared-representation account to the SiCE (Schwarz & Heinze, 1998).

## Experiment 2

We conducted Experiment 2 to bolster the channels-based interpretation discussed above. This experiment was identical to Experiment 1, except for two important changes:First, in Experiment 2 the digit “2” was set as the reference digit in the different condition; second, the identical condition presented a pair of 5s (i.e., 5-5). Corresponding with these two changes, we had two hypotheses. First, that this time the PSE of the reference digit (i.e., “2”) in the different condition would be larger than the PSE of the reference digit (i.e., “5”) in the identical condition. This is expected because the physical magnitude of the reference digit in the different condition should compensate for its smaller numerical magnitude. Second, we hypothesized that the mean PSEs of the identical condition would not be different between experiments.

### Method

#### Participants

We recruited 20 participants (14 females, mean age = 26.6 years, *SD* = 2.25) from the BGU participant pool. These participants did not take part in Experiment 1. Participants received monetary reward for their participation. All participants had normal or corrected-to-normal vision. All participants signed a consent form prior to their participation in the experiment. Participants were debriefed in the end of the experiment.

#### Stimuli

The stimuli and their sizes were identical to Experiment 1. However, in Experiment 2 the pairs we used were 5-5 and 2-5. The standard digit was always “5”.

#### Design and procedure

The design and procedure in Experiment 2 were identical to Experiment 1.

### Results

The preprocessing of the data was identical to Experiment 1. First, we excluded trials in which participants failed to respond (consisting of 0.6% of all trials). Then, we fitted a sigmoid function to the data and computed GOF scores, for each condition. Two participants were excluded due to low GOF scores (0.47 and 0.33). After their exclusion, the total averages (*SD*s) of GOF scores were 0.95 (0.03) and 0.94 (0.04) for the identical and different conditions, respectively. We calculated the PSE and just noticeable difference (JND) of each participant, in each condition. We excluded two participants: one participant whose PSE deviated by 2.51 *SD*s from the mean PSE in the different condition, and another participant whose JND deviated by 2.64 *SD*s from the mean JND in the different condition. Thus, further analyses were performed on data of 16 participants^3^. We conducted two t-tests to examine the difference between the PSE means and the JND means between conditions. We found that the mean PSE in the identical condition was significantly smaller than the mean PSE in the different condition, $95\% {\text{CI}}\left[-1.93, -0.94\right], t\left(15\right)=-6.17, p<.001, d=-1.59.$ In contrast, JND means were not significantly different between conditions ($t\left(15\right)=-1.48, p=.15$). Finally, we conducted two t-tests to compare the PSE and JND of the identical condition between experiments. Our tests indicated that neither the PSE means ($t\left(30\right)=0.4, p=.68$), nor the JND means ($t\left(30\right)=1.18, p=.24$), were significantly different between experiments. Figure 1 presents the psychophysical functions of all four experimental conditions. Figure 2 presents PSE and JND means of all four experimental conditions.

#### Physical magnitude compensates for numerical magnitude

Experiments 1 and 2 indicated that physical magnitude compensated for differences in numerical magnitudes to perceptually match two digits. To quantify this compensation (in percentages), we applied the following calculation: $\frac{{{\text{PSE}}}_{diff}-{{\text{PSE}}}_{ident}}{{{\text{PSE}}}_{ident}}\bullet 100$. In Experiment 1, the physical magnitude of “5” (i.e., the reference digit in the different condition) needed to be reduced by 0.88% (on average, SE = 0.36%) to perceptually match a “2”. In Experiment 2, the physical magnitude of “2” (i.e., the reference digit in the different condition) needed to be enlarged by 2.06% (on average, SE = 0.33%) to perceptually match a “5”.

### Discussion

In two psychophysical experiments, we examined the interaction between physical and numerical magnitude of digits. Our methodology controlled for physical characteristics (i.e., the configuration of line strokes that make up a digit), so any modulation would be attributed to differences in numerical magnitude. We observed that participants overestimated the physical size of a numerically larger digit (Experiment 1), whereas the opposite was true for a numerically smaller digit (Experiment 2). Our results expand on the interaction between physical and numerical magnitude of digits on the perceptual level. We suggest that participants constructed a-modal mental representations of digits that received inputs from both physical and numerical channels of magnitude. Thus, to perceptually match digits, participants needed larger (or smaller) physical magnitude to compensate for smaller (or larger) numerical magnitude. To the best of our knowledge, our study is a pioneer in quantifying numerical (i.e., conceptual) and physical magnitude interaction in perceptual estimations (see Harris et al., 2016, for a similar approach to quantifying how perceived emotion from faces is influenced by gender-related attributes).

Our results fit well with the shared-representation account of the SiCE (Schwarz & Heinze, 1998). Our methodology allowed us to quantify the influence of the irrelevant numerical value on physical size judgements. According to the shared-representation account of the SiCE, the under or over estimation is due to changes in the mental representation. These changes are caused by the effect of the irrelevant information (Reike & Schwarz, 2017). Similar to visual illusions (e.g., the Delboeuf illusion), we suggest that perception reflects changes in the mental representation (Helmholtz, 1896). This quantification expands the descriptive and interpretive power of the shared-representation account of the SiCE. Quantifying how numerical and physical magnitudes interact on the perceptual level allows to delineate (rather than simply indicate) how magnitude channels converge on a single mental representation. Significantly, under a more nuanced shared-representation account of the SiCE, novel investigations of our symbolic numerical system could ensue. For instance, a quantifiable measure could help evaluate the development of our symbolic numerical system in children throughout school years. Moreover, it could illuminate why individuals with developmental dyscalculia fail to recruit the symbolic numerical system when needed, and whether these failures are related to the mental representations comprising this system.

Note that our interpretation of the modulations we observed is attributed to perceptual mechanisms that operate in early, stimulus-evaluation stages. However, there remains a possibility that participants’ responses gave rise to these modulations. In other words, it is possible that the modulations of the PSE reflect (to some degree) response-related mechanisms that operate later in processing. For instance, it may be that under ambiguous size differences, participants reverted to reporting the numerically larger digit (i.e., 5) instead of the physically larger digit. It is possible that the numerically larger digit (i.e., “5”) attracted more attention than its numerically smaller counterpart (i.e., “2”; Krause et al., 2017). However, it does not necessarily mean that such attentional bias subsequently gave rise to biased responses. In fact, it was previously shown that response-related mechanisms were not affected by numerical magnitude whereas perceptual mechanisms were affected by it (Reike & Schwarz, 2017). Accordingly, we opt for the representation-based interpretation we put forward here. Note that this interpretation is further supported by evidence of early numerical processing (e.g., Krause et al., 2017) and the conventional, perception-based interpretation of the PSE (whose modulations constitute our key findings).

Interestingly, we observed that participants underestimated the size of a numerically smaller digit (Experiment 2) to a larger extent than they overestimated the size of a numerically larger digit (Experiment 1). This is demonstrated by the difference between the gray bars (Experiment 2) compared with the difference between the white bars (Experiment 1) in Panel A of Fig. 2. To interpret, the digit “2” in Experiment 2 needed to be enlarged by 2.06% (on average) to be perceived as equal in physical terms to a “5”, whereas the digit “5” in Experiment 1 needed a to be reduced by 0.88% (on average) to be perceived as equal in physical terms to a “2”. We did not anticipate this asymmetry between experiments. However, we surmise that this difference may reflect a difference in numerical processing between experiments. For instance, it may be that in Experiment 2, in which the digit “5” was repeatedly presented, stronger numerical processing ensued because participants were instructed to report the physically larger digit. Put differently, reporting the physically larger digit in the context of 5s (Experiment 2) may have given rise to stronger numerical processing than in the context of 2s (Experiment 1) (see Krause et al., 2017; Risko et al., 2013 for similar discussions about contextual influences on numerical processing of digits). However, this post hoc suggestion is only provisional. Future studies may shed more light on what this difference underlies.

On a final note, we highlight that our psychophysical methodology could also be applied to study interactions between other channels of magnitudes (e.g., numerical magnitude and luminance; Walsh, 2003). This would allow examining whether channels of magnitude operate similarly across all magnitudes, or that some magnitudes interact more closely (or remotely) on the perceptual level.

## Figures and Tables

**Fig. 3:**
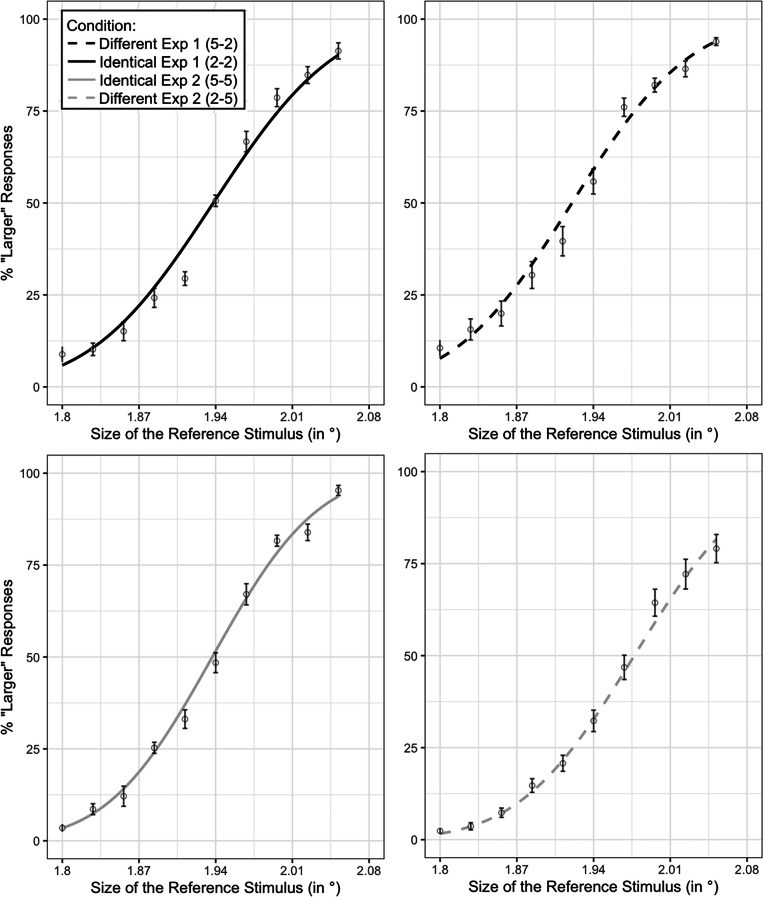
Estimated probabilities of size of reference stimulus by condition. *Note.* The figure presents the estimated probabilities of each size of reference stimulus by condition. The legend denotes the condition. The left digit in the legend labels denotes the reference digit in each condition (e.g., “5” was the reference digit in the different condition in Experiment 1). The X-axis denotes the size of the reference digit in visual angles. The Y-axis denotes the probability to respond “larger” to the reference digit. White data points correspond to the 10 sizes used in the experiments, denoting estimated mean probabilities to respond “larger”. Error bars denote standard errors of estimated mean probabilities (SE)

## Data Availability

The data and materials for our experiments are available via the Open Science Framework at https://osf. io/t5qjs/. These experiments were not preregistered.

## Appendix

Appendix Fig. 3

## Footnotes

1 Due to a technical issue, a reference digit identical in size to the standard digit was displayed. As a result, there was asymmetry between the number of smaller size values and larger size values.

2 Due to a technical issue, the sizes 1.84 ^∘^ and 2.0 ^∘^ were repeated 16 times in each experimental block. Note that this was true for all conditions, in both experiments.

3 As per the request of an anonymous reviewer, we re-ran the same analyses on all participants in Experiment 2 (including the two participants we originally excluded due to deviant parameter values). Note that their inclusion did not change our results: the mean PSE in the identical condition was significantly smaller than the mean PSE in the different condition, $95\% {\text{CI}}\left[-2.24, -1.10\right], t\left(17\right)=-6.21, p<.001, d=-1.51$. In contrast to the PSE (and similar to the results without these two participants), JND means were not significantly different between conditions ($t\left(17\right)=-1.77, p=.09$).
